# A Novel PLP-Dependent Alanine/Serine Racemase From the Hyperthermophilic Archaeon *Pyrococcus horikoshii* OT-3

**DOI:** 10.3389/fmicb.2018.01481

**Published:** 2018-07-09

**Authors:** Ryushi Kawakami, Tatsuya Ohshida, Haruhiko Sakuraba, Toshihisa Ohshima

**Affiliations:** ^1^Division of Bioscience and Bioindustry, Graduate School of Technology, Industrial and Social Sciences, Tokushima University, Tokushima, Japan; ^2^Department of Applied Biological Science, Faculty of Agriculture, Kagawa University, Kagawa, Japan; ^3^Department of Biomedical Engineering, Faculty of Engineering, Osaka Institute of Technology, Osaka, Japan

**Keywords:** Ala racemase, Ser racemase, PLP-dependent enzyme, hyperthermophilic archaea, *Pyrococcus horikoshii* OT-3, D-amino acid

## Abstract

We recently identified and characterized a novel broad substrate specificity amino acid racemase (BAR) from the hyperthermophilic archaeon *Pyrococcus horikoshii* OT-3. Three genes, *PH0782*, *PH1423*, and *PH1501*, encoding homologs exhibiting about 45% sequence identity with BAR were present in the *P. horikoshii* genome. In this study, we detected pyridoxal 5′-phosphate (PLP)-dependent amino acid racemase activity in the protein encoded by *PH0782*. The enzyme showed activity toward Ala, Ser, Thr, and Val, but the catalytic efficiency with Thr or Val was much lower than with Ala or Ser. The enzyme was therefore designated Ala/Ser racemase (ASR). Like BAR, ASR was highly stable at high temperatures and over a wide range of pHs, though its hexameric structure differed from the dimeric structure of BAR. No activity was detected in K291A or D234A ASR mutants. This suggests that, as in Ile 2-epimerase (ILEP) from *Lactobacillus buchneri* JCM1115, these residues are involved in Schiff base formation and substrate interaction, respectively. Unlike BAR, enhanced ASR activity was not detected in *P. horikoshii* cells cultivated in the presence of D-Ala or D-Ser. This is the first description of a PLP-dependent fold type I ASR in archaea.

## Introduction

D-Amino acids play a variety of important roles in many organisms. In mammals, for example, D-Ser and D-Asp are known to be *N*-methyl-D-Asp (NMDA) receptor co-agonists (Errico et al., 2015), while D-Ala is a major osmolyte responsible for intracellular isosmotic regulation in the tissues of bivalve and kuruma prawn (Abe et al., 2005; Yoshikawa and Yokoyama, 2015). In bacteria, it is well known thatD-Ala and D-Glu are components of the peptidoglycan cell wall (Hancock, 1960; Ghuysen, 1961), as are D-Ser and D-Asp (Reynolds and Courvalin, 2005; Veiga et al., 2006). Although free D-amino acids such as D-Asp, D-Ala, and D-Ser have also been detected in hyperthermophilic archaea (Matsumoto et al., 1999; Nagata et al., 1999; Long et al., 2001), the cell walls of archaea such as the *Pyrococcus* and *Thermococcus* species are S-layers, composed of hexagonally or tetragonally arranged proteins or glycoproteins and do not contain D-amino acids. The physiological function of D-amino acids in archaeal cells thus remains unclear.

Free D-amino acids in cells are generally produced by amino acid racemases, which catalyze pyridoxal 5′-phosphate (PLP)-dependent or PLP-independent racemization of amino acids. PLP-independent Asp racemases (AspRs) and PLP-dependent AlaR and SerR have been found in archaeal cells and characterized (Matsumoto et al., 1999; Long et al., 2001; Moore and Leigh, 2005; Ohnishi et al., 2008; Aihara et al., 2016; Washio et al., 2016). This includes detailed structural and functional characterization of a PLP-independent AspR from the hyperthermophilic archaeon *Pyrococcus horikoshii* OT-3 (Liu et al., 2001, 2002a,b; Yoshida et al., 2006; Ohtaki et al., 2008; Kita et al., 2009). We recently detected novel PLP-dependent amino acid racemase activity toward Met, Leu, and Phe in the crude extract of *P. horikoshii*, identified the enzyme gene (ORF ID: *PH0138*), and determined the properties of the recombinant protein (Kawakami et al., 2015, 2017). That enzyme showed activity toward 10 amino acids and was therefore named BAR.

This enzyme was originally annotated as a GABA-AT in the genome database, and its primary structure is consistent with a fold-type I PLP-dependent enzyme. Three other genes within the *P. horikoshii* genome, *PH0782*, *PH1423*, and *PH1501*, encode proteins that are also annotated as GABA-ATs and exhibit high similarity to BAR. This suggests these homologs also function as amino acid racemases. We previously reported that BAR activity is markedly increased in *P. horikoshii* cells grown on medium supplemented with D-*allo*-Ile, which suggests BAR mediates utilization of D-amino acids for growth (Kawakami et al., 2015). In the present study, we constructed expression systems for BAR homologs using pET vector and detected amino acid racemase activity toward Ala and Ser in the recombinant PH0782 enzyme. We then characterized the enzyme and identified the residues responsible for its catalytic activity. To better understand the physiological function of this enzyme, we examined the level of AlaR activity in *P. horikoshii* cells under various growth conditions.

## Materials and Methods

### Materials

*o*-Phthalaldehyde and *N*-*tert*-butyloxycarbonyl-L-cysteine were from Wako (Osaka, Japan) and Sigma–Aldrich (Tokyo, Japan), respectively. D- and L-amino acids were from Wako and Tokyo Chemical Industry (Tokyo). All other chemicals were of reagent grade.

### Construction of Expression Plasmids for BAR Homologs

In this study, we constructed expression plasmids for *PH0782*, *PH1423*, and *PH1501* genes using a pET system. The three genes were amplified using PCR with *P. horikoshii* genomic DNA as the template. The primer sets used in the PCR are listed in Supplementary Table S1. Forward and reverse primers introduced an *Nde*I site overlapping the 5′ initiation codon and a *Bgl*II (for *PH0782*) or *Bam*HI (for *PH1423* and *PH1501*) site proximal to the 3′ end of the termination codon. PCR reactions were run using PrimeStar Max DNA polymerase (Takara, Tokyo) according to the manufacturer’s instructions. Amplified fragments were purified, introduced into pCR4-TOPO (Invitrogen, Tokyo) and sequenced. The resultant TOPO/*PH0782*, TOPO/*PH1423* and TOPO/*PH1501* were digested with *Nde*I and *Bam*HI (*Bgl*II for *PH0782*) and introduced into pET11a (Novagen, Tokyo) to generate pET11a/*PH0782*, pET11a/*PH1423*, and pET11a/*PH1501*, respectively.

For construction of the K291A and D234A mutants, two sets of primers were designed (Supplementary Table S1) and pET11a/*PH0782* was used as the template. The non-PCR reaction was run with PrimeStar Max DNA polymerase (Takara, Tokyo) using the standard protocol supplied by the manufacturer. The restriction enzyme *Dpn*I was added to the reaction mixture to digest the template DNA. An aliquot of the reaction mixture was then used to transform TOP 10 cells (Stratagene, Tokyo). To screen for the correct mutation, the plasmids were extracted from the transformants and whole gene sequencing was conducted using a genetic analyzer (Model 3130, Applied Biosystems, Tokyo).

### Expression and Purification of Recombinant Enzymes

The procedures used to express and purify PH0782 were similar to those used for BAR (Kawakami et al., 2017), except that *Escherichia coli* BL21 (DE3) cells (Stratagene) were used as the competent cells. For analysis of the mutant enzymes and substrate screening, enzymes partially purified through heat treatment at 90°C for 20 min were used.

Protein concentrations were determined using the Bradford method (Bradford, 1976). Bovine serum albumin served as the standard.

### Subunit and Native Molecular Mass Determination

SDS–PAGE (Laemmli, 1970) was used to determine the subunit molecular mass of the enzymes. Myosin (200 kDa), β-galactosidase (116.3 kDa), phosphorylase B (97.4 kDa), serum albumin (66.2 kDa), ovalbumin (45 kDa), carbonic anhydrase (31 kDa), trypsin inhibitor (21.5 kDa), lysozyme (14.4 kDa), and aprotinin (6.5 kDa) were used as molecular mass standards (Bio-Rad, Tokyo). The native molecular mass of PH0782 was determined using gel filtration chromatography with a HiLoad 26/60 Superdex 200 column (GE Healthcare, Tokyo). Thyroglobulin (670 kDa), γ-globulin (158 kDa), Ovalbumin (44 kDa), and Myoglobin (17 kDa) were used as molecular mass standards (Bio-Rad).

### Separation and Determination of DL-Amino Acids Using UPLC

UPLC analyses were performed for quantitative determination of L- or D-amino acids produced in the enzyme assays used for substrate screening, assessment of pH dependency, and kinetic analysis. For the enzyme assays, the standard reaction mixture included 100 mM HEPES (pH 7.0), 10 mM L-amino acid, 0.04 mM PLP, and 1 μg of enzyme in a 100-μL volume, which was incubated at 80°C for 30 min. After incubation, the reaction mixture was immediately cooled, 6% trichloroacetic acid was added, and the precipitate was removed by centrifugation. The supernatant was then neutralized using NaOH, and the amino acids in the mixture were derivatized with OPA and NBC. In the screening assays, the derivatized amino acids were analyzed using an X-pressPak V-C18 column (2.0 mm by 50 mm, Jasco, Tokyo) as described previously (Kawakami et al., 2017). For simultaneous kinetics analysis using Ala, Ser, Val, and Thr as substrates, citrate solutions (5 mM, pH5.8) in 15 and 60% ethanol were used as buffers A and B, respectively, and the gradient program was operated as follows: 0–20% B for 4.5 min, 20–40% B for 2.5 min, 40–70% B for 1.0 min, 70% B for 0.5 min, and 70–0% B for 0.5 min.

### Characterization of Enzymes

For substrate screening, 10 μg of each enzyme were added to the reaction mixture and incubated for 60 min. Ala, Val, Leu, Ile, Phe, Met, Trp, Tyr, Ser, Thr, Asn, Gln, and Arg were used as substrates. To determine pH dependency, L-Ala was used as the substrate and the activity was assayed under various buffer conditions (acetate [pH 5.5–6.5], MES [pH 5.5–7.0], phosphate [pH 6.0–8.0], HEPES [pH 6.5–8.5], and CHES [pH 8.5–9.5]; buffer pH was adjusted at 25°C). To determine the kinetic parameters, racemase activity was assayed in the presence of 1–20 mM Ala or Ser (*n* = 3). Reaction rates were independently calculated, and apparent *V*_max_ and *K*_m_ values were analyzed with Prism 5.0 (GraphPad Software) using a non-linear regression model.

To assess the pH stability of PH0782, the enzyme (0.1 mg/ml) was incubated at 80°C for 2 h in several buffers (100 mM, acetate [pH 4.0–6.0], phosphate [pH 7.0–8.0], glycine [pH 9.0–10.0], and phosphate [pH 11.0–12.0]), and the residual activity was assayed. The thermostability of PH0782 was assessed by determining the residual activity after incubation at selected temperatures for 30 min and at 80°C for several different incubation times. The residual activities in the temperature and pH stability assays were determined spectrophotometrically using L-Ala as the substrate, as described previously (Kawakami et al., 2015).

### Cultivation of *P. horikoshii* Under Various Conditions and Determination of Racemase Activities

The activity of BAR was greatly increased in *P. horikoshii* cells grown on medium supplemented with D-*allo*-Ile (Kawakami et al., 2015). We therefore investigated ASR activity in *P. horikoshii* cells grown on medium supplemented with D-Ala, D-Ser or D-*allo*-Ile. Cultivation of *P. horikoshii* OT-3 with a D-amino acid was previously described by Kawakami et al. (2015).

In the kuruma prawn, D-Ala produced from L-Ala by AlaR reportedly functions as an osmolyte responsible for intracellular isosmotic regulation (Yoshikawa and Yokoyama, 2015). To determine whether ASR is responsible for increasing the NaCl concentration in the medium, *P. horikoshii* was cultivated for 3 h at several NaCl concentrations (1.5, 2.5, 3.5, and 4.5%) in standard medium after cultivation for 18 h at 90°C in standard medium (2.5% NaCl). The *P. horikoshii* cells collected by centrifugation were disrupted by sonication, after which the extracts were cleared through another centrifugation, and the supernatants were used for determination of racemase activity. Spectrophotometric assays were performed as described previously (Kawakami et al., 2015), and L-Met and L-Ala were used as substrates for BAR and ASR, respectively.

## Results

### Screening of Substrates for Racemase Activity of BAR Homologs

*Escherichia coli* BL21 (DE3) cells harboring expression plasmids encoding BAR homologs (pET11a/*PH0782*, pET11a/*PH1423*, and pET11a/*PH1501*) were grown in LB medium, and gene expression was induced using IPTG. The cells were then disrupted by sonication, and the resultant supernatants were heated at 90°C for 20 min in the presence of 0.1M citrate (pH 5.5) buffer. The heat-treated supernatants were then subjected to SDS–PAGE, and bands derived from BAR homologs were clearly detected at about 50 kDa (data not shown). To screen for amino acid racemase activity, L-forms of Ala, Val, Leu, Ile, Phe, Met, Trp, Tyr, Ser, Thr, Asn, Gln, and Arg were used as substrates with the BAR homologs, and the reaction mixtures were analyzed using UPLC. Peaks for the D-forms of Ala and Ser were clearly detected in the reaction mixture containing PH0782. Weaker peaks for D-Val and D-*allo*-Thr were also detected with PH0782 (data not shown). Slight peaks for the D-forms of Met, Phe, and Leu were detected in the reaction mixture containing PH1501. No peak corresponding to a D-amino acid was detected in the reaction mixture containing PH1423 (data not shown). In addition, spectrophotometric assay revealed no racemase activity toward Pro in any of the enzymes (data not shown).

### Purification and Molecular Mass Determination of PH0782

**FIGURE 1:**
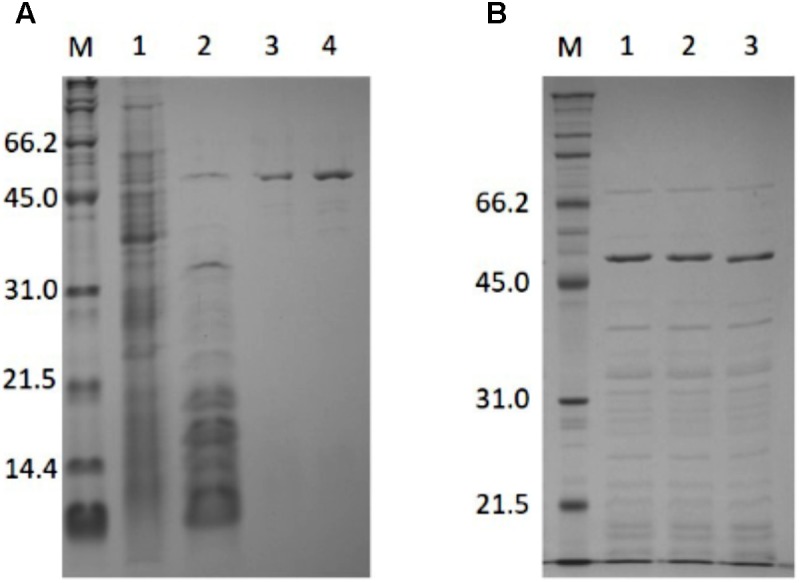
SDS–PAGE analysis of the purification of PH0782 **(A)** and its mutants **(B)**. **(A)** 15% polyacrylamide gel was used for the analysis. Lanes: M, markers; 1, crude extract (30 μg); 2, heat treatment (6.5 μg); 3, butyl-Toyopearl (1.4 μg); and 4, DEAE-cellurofine (1.4 μg). **(B)** Crude extracts of cells expressing wild type and mutant PH0782 were heated at 90°C for 20 min with 0.1M citrate (pH 5.5), and the supernatants (each of about 7 μg) were applied to 12% polyacrylamide gel. Lanes: M, markers; 1, wild type; 2, K291A mutant; and 3, D234A mutant.

PH0782 was successfully purified using the same procedure used for BAR (**Figure 1A**). About 4 mg of purified enzyme were obtained from 3.2 L of LB medium. SDS–PAGE showed the subunit molecular mass of purified PH0782 to be about 54.9 kDa, which approximates the molecular mass (50.5 kDa) calculated from the primary structure. Using gel filtration chromatography, the native molecular mass of PH0782 was estimated to be about 300 kDa, which suggests the enzyme exists as a hexamer.

### Characterization of PH0782

The activity of the purified enzyme was doubled by the addition of PLP and completely inhibited by the addition of 1 mM hydroxylamine hydrochloride, a known inhibitor of PLP-dependent enzymes, suggesting PLP is a cofactor. To prepare an apo-protein of PH0782, the enzyme was incubated at 80°C for 18 h in the assay mixture. The apo-enzyme exhibited no activity in the absence of PLP and recovered its activity by addition of PLP.

**FIGURE 2:**
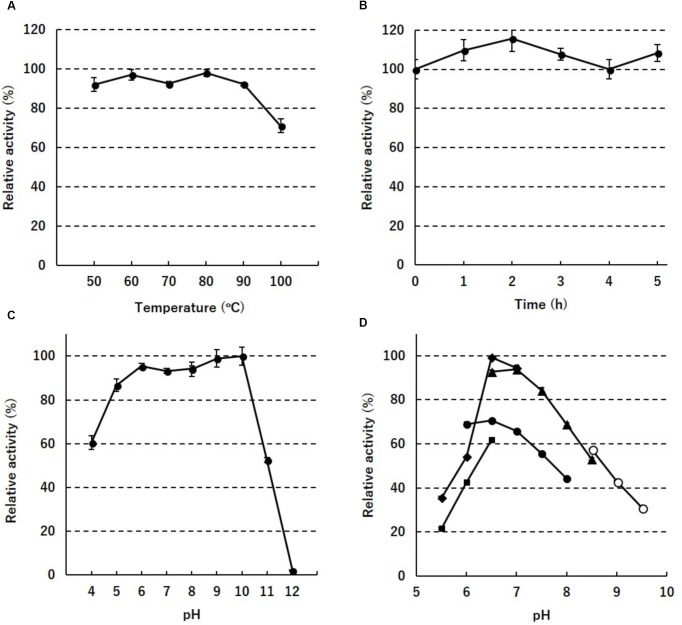
Enzyme stability against temperature **(A)**, time **(B)**, and pH **(C)**, and the pH dependency **(D)** of PH0782. In **(D)**, acetate (squares), MES (diamonds), phosphate (closed circles), HEPES (triangles), and CHES (open circles) were used as buffers.

To assess the thermostability of PH0782, its enzyme activity was determined after incubation at different temperatures for various times. When the enzyme was incubated for 30 min at temperatures between 50 and 100°C, full enzyme activity was retained until 90°C, but about 30% was lost at 100°C (**Figure 2A**). When the enzyme was incubated at 80°C, full activity was retained for at least 5 h (**Figure 2B**). When the enzyme was incubated at 80°C for 2 h at various pHs, full enzyme activity was retained at pHs ranging from 6 to 10 (**Figure 2C**). To determine the optimal pH for enzyme activity, the assay was performed at pHs ranging from 5.5 to 9.5. Highest activity was detected at around pH 6.5–7.0 in MES and HEPES buffers (**Figure 2D**).

To determine the kinetic parameters of PH0782 enzyme activity toward Ala and Ser, the initial velocities of both D-amino acid formation and L-amino acid formation were measured at concentrations ranging from 1 to 20 mM. The relation between substrate concentration and initial velocity fitted well into a non-linear regression model. The calculated *V*_max_ and *K*_m_ values for PH0782 are shown in **Table 1**. The *V*_max_ values toward D-Ala (49.5 ± 2.09 μmol/min/mg) and D-Ser (41.2 ± 1.10 μmol/min/mg) were 1.7 and 1.3 times higher than those toward L-Ala (28.2 ± 0.76 μmol/min/mg) and L-Ser (31.4 ± 0.80 μmol/min/mg), respectively. The *K*_m_ values for L-Ala (3.3 ± 0.22 mM) and L-Ser (4.5 ± 0.32 mM) were lower than those for D-Ala (5.5 ± 0.59 mM) and D-Ser (5.9 ± 0.40 mM), respectively. The kinetic parameters for Val and Thr were not determined because of the high *K*_m_ and/or low *V*_max_ predicted from the preliminary analysis.

**Table 1:** Kinetic analysis of the amino acid racemase activity of PH0782.

Substrate	*V*_max_ (μmol/min/mg)	*K*_m_ (mM)	*k*_cat_/*K*_m_ (/s/mM)
L-Ala	28.2 ± 0.76	3.3 ± 0.22	7.19 ± 0.52
L-Ser	31.4 ± 0.80	4.5 ± 0.32	5.87 ± 0.44
D-Ala	49.5 ± 2.09	5.5 ± 0.59	7.58 ± 0.87
D-Ser	41.2 ± 1.10	5.9 ± 0.40	5.88 ± 0.43


### Mutation Analysis of the Catalytic Residues

**FIGURE 3:**
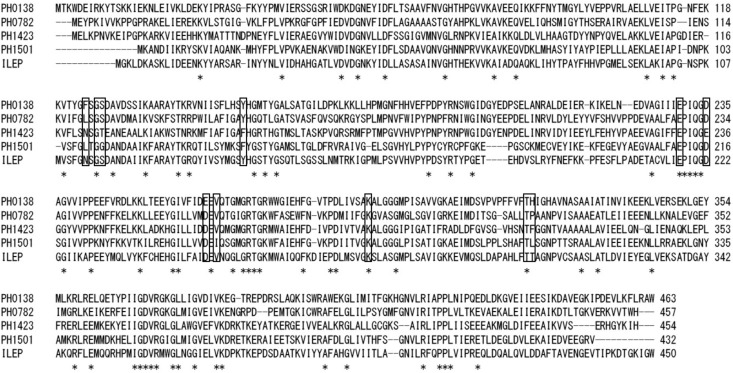
Sequence alignment of BAR homologs and ILEP from *L. buchneri*. Conserved residues are indicated by asterisks. Residues interacting with PLP, which were determined from the structure of ILEP (Hayashi et al., 2017), are boxed.

Two PH0782 mutants (K291A and D234A) were constructed to confirm that these residues are important for the enzyme’s catalytic activity. **Figure 3** shows an alignment of the primary structures of BAR homologs and ILEP from *Lactobacillus buchneri* JCM1115 (Mutaguchi et al., 2013). The crystal structure of ILEP revealed that K280 and D222 are responsible for the interaction with PLP (Hayashi et al., 2017). These residues corresponded to residues K291 and D234 in PH0782 (**Figure 3**). After expression in *E. coli*, the mutant proteins were clearly observed on SDS–PAGE after heat treatment at 90°C (**Figure 1B**). Spectrophotometric assay using L-Ala as a substrate detected no activity with either the K291A or D234A mutant.

### AlaR Activity in *P. horikoshii* Cells Grown Under Various Conditions

To determine whether ASR mediates utilization of D-amino acids for growth, *P. horikoshii* was cultivated in medium containing several D-amino acids. The activity toward Met derived from BAR was detected in all *P. horikoshii* cells tested. As previously described, the highest activity was detected in cells grown on medium containing D-*allo*-Ile (Kawakami et al., 2015). By contrast, no AlaR activity was detected in any of the tested cells.

Alanine racemase activity in *P. horikoshii* cells cultivated at several NaCl concentrations (1.5, 2.5, 3.5, and 4.5%) was determined to test whether ASR is responsible for increasing the NaCl concentration in the medium, but no activity was detected in any of the tested cells.

## Discussion

In an earlier study, we revealed that PH0138, which was originally annotated as a putative GABA-AT, catalyzed the racemization of a number of hydrophobic and aromatic amino acids. We therefore designated the enzyme a BAR (Kawakami et al., 2015). In addition, we found that three other genes, *PH0782*, *PH1423*, and *PH1501*, were also annotated as putative GABA-AT genes and had high sequence similarity with BAR (about 45%). In the present study, we succeeded in constructing expression plasmids encoding these genes and found that PH0782 has amino acid racemase activity toward Ala, Ser, Val, and Thr. The racemase activity was completely inhibited by hydroxylamine hydrochloride, but strangely, we did not succeed to prepare apo-protein of PH0782 by dialysis against the buffer containing hydroxylamine hydrochloride. In addition, apo-protein was not obtained even after incubation at 80°C for 18 h with or without hydroxylamine. This suggests that PLP binds tightly to PH0782 and is stabilized in the enzyme complex, although free PLP is known to be thermolabile. On the other hand, after the enzyme was incubated at 80°C for 18 h in the assay mixture containing L-Ala as substrate, it showed no activity but recovered its activity by the addition of PLP. This indicates that apo-protein is probably prepared by incubation in the presence of substrate at 80°C. But further studies might be needed to clarify these observations. The enzyme activity of PH0782 produced equimolar amounts of L-Ala and D-Ala and of L-Ser and D-Ser, and the equilibrium constant for racemization (*K*_eq_) was determined to be nearly 1 (Supplementary Figure S1). These results indicate that PH0782 catalyzes the racemization reaction. Kinetic analysis revealed that the catalytic efficiency with Ala (7.19 ± 0.52 /S/MM FOR L-Ala and 7.58 ± 0.87 /s/mM for D-Ala) is comparable to that with Ser (5.87 ± 0.44 /s/mM for L-Ser and 5.88 ± 0.43 /s/mM for D-Ser), but the efficiency was much lower with Val or Thr. We therefore conclude that PH0782 is an ASR, not a BAR. Further analyses will be necessary to identify the substrates and activities of PH1423 and PH1501, as they exhibited little or no activity in the present study.

Alanine racemases and serine racemases are well characterized as PLP-dependent racemases (Yoshimura and Esaki, 2003; Radkov and Moe, 2014; Hernández and Cava, 2016) that generally show strict substrate specificity for Ala and Ser, and are classified as fold type III and type II PLP-dependent enzymes, respectively. By contrast, ASR shows equivalent substrate specificity for Ala and Ser and is classified as fold type I. Although racemase activities toward Ala and Ser have been detected in extract from the hyperthermophilic archaeon *Pyrobaculum islandicum*, the details remain unclear (Nagata et al., 2002). Recently, ILEP from *L. buchneri* was found to share 37 and 40% sequence identity with ASR and BAR, respectively, and to be a fold type I enzyme that catalyzes the epimerization and/or racemization of multiple hydrophobic amino acids, including Ile and Val (Mutaguchi et al., 2013). More recently, the crystal structures of ILEP in its apo-form and in complex with PLP, PLP-L-Ile, and PLP-D-*allo*-Ile were determined at resolutions of 1.94–2.77 Å (Hayashi et al., 2017). The residues in ILEP that interact with PLP are well conserved in both ASR and BAR (**Figure 3**). Among these residues, K280 (K291 in ASR) forms an internal aldimine (Schiff-base) linkage with PLP, and D222 (D234) is the most proximate to the Cα atom of D-*allo*-Ile. In addition, the D222A and D222N ILEP mutants exhibit almost no activity (Hayashi et al., 2017). Likewise, in the present study, the K291A and D234A ASR mutants exhibited no activity. These results suggest that, as in ILEP, these residues are involved in Schiff base formation and substrate interaction, and that the monomer structures of ASR and BAR are similar to that of ILEP, though the quaternary structures differ among the three enzymes: BAR is dimeric, ILEP is tetrameric, and ASR is hexameric.

Several free D-amino acids, including D-Asp and D-Ala, are present within the cells of hyperthermophilic archaea, but their functions remain unknown. The presence of ASR in *P. horikoshii* suggests that D-Ala and/or D-Ser serve some function in these cells. We previously observed that cellular BAR activity in *P. horikoshii* is dramatically enhanced when the cells are grown on medium supplemented with D-*allo*-Ile (Kawakami et al., 2015). This was the first clue that D-amino acids are involved with expression of enzyme genes in hyperthermophilic archaea. We therefore investigated ASR activity in *P. horikoshii* grown on medium supplemented with D-amino acids; however, no activity was detected in any of the cells tested. It is also known that D-Ala acts as an osmolyte to protect tissues in crustaceans from changes in osmotic pressure (Yoshikawa and Yokoyama, 2015). We therefore investigated ASR activity in *P. horikoshii* cells grown in the presence of high and low salt concentrations, but again no activity was detected. Although the physiological function of ASR remains unclear, the ASR-encoding *PH0782* gene forms a cluster with *PH0781* gene, which encodes a putative alanine transport protein, and this gene cluster is widely distributed in other *Pyrococcus* and *Thermococcus* strains. This suggests the enzyme may function in D-Ala production from L-Ala in these strains.

## Author Contributions

RK conceived and designed the study, collected, analyzed and interpreted the data, and drafted the article. TAO carried out, analyzed, and interpreted a pair of experiments. HS and TOO interpreted data and helped draft the manuscript.

## Conflict of Interest Statement

The authors declare that the research was conducted in the absence of any commercial or financial relationships that could be construed as a potential conflict of interest.

## Abbreviations

AlaR: alanine racemase
ASR: Ala/Ser racemase
BAR: broad substrate specificity amino acid racemase
GABA-AT: γ-aminobutyric acid aminotransferase
IPTG: isopropyl-β-D-thiogalactopyranoside
NBC: *N*-*tert*-butyloxycarbonyl-L-cystein
OPA: *o*-phthalaldehyde
SerR: serine racemase
