# Supplementary material for: A Novel PLP-Dependent Alanine/Serine Racemase From the Hyperthermophilic Archaeon Pyrococcus horikoshii OT-3
Source: Front Microbiol. 2018 Jul 9;9:1481. doi: 10.3389/fmicb.2018.01481 (PMC6047364; doi:10.3389/fmicb.2018.01481)

**Supplementary Table S1 Sequences of primers used in this study**

| Primer | Sequence |
| --- | --- |
| PH0782-NdeI | 5’-CCATATGGAGTACCCAAAGATAGTTG-3’ |
| PH0782-BglII | 5’-CCAGATCTTCAGTGCCAAGTTACTAC-3’ |
| PH1423-NdeI | 5’-CCATATGGAGTTGAAGCCAAACGTTAAAG-3’ |
| PH1423-BamHI | 5’-TGGATCCCTAATGAATCTTGTATCCGTGC-3’ |
| PH1501-NdeI | 5’-CCATATGAAGGCCAATGACATAATTAAG-3’ |
| PH1501-BamHI | 5’-TTGGATCCTCATACTCGGCCTTCTTCAAC-3’ |
| PH0782(K291A)Fw | 5’-TTTGGGGCAGGAGTAGCGAGTGGAATG-3’ |
| PH0782(K291A)Rv | 5’-TACTCCTGCCCCAAATATTATCATGTC-3’ |
| PH0782(D234A)Fw | 5’-CAGGGAGCGGCAGGGATCGTTGTCCCT-3’ |
| PH0782(D234A)Rv | 5’-CCCTGCCGCTCCCTGGATTGGTTCAGC-3’ |

**Supplementary Fig. S1 Time courses of the enzyme reaction with substrates**

The enzyme (1 μg) was incubated at 80 °C with 10 mM each of l-Ala (A), d-Ala (B), l-Ser (C), and d-Ser (D) as substrates in a total reaction volume of 0.1 ml. The enzyme reaction was stopped at appropriate time and the substrate and product concentrations were determined by UPLC assay. The data for l-Ala, d-Ala, l-Ser, and d-Ser were depicted as triangles, squares, diamonds, and circles, respectively.


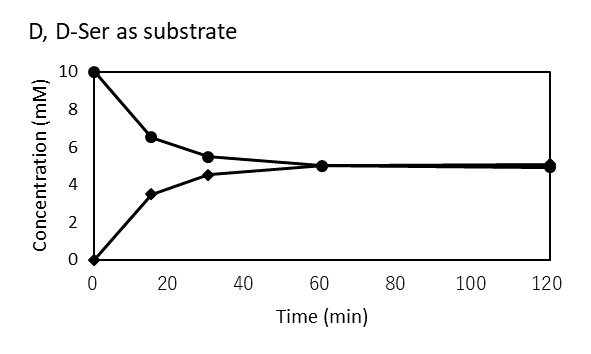

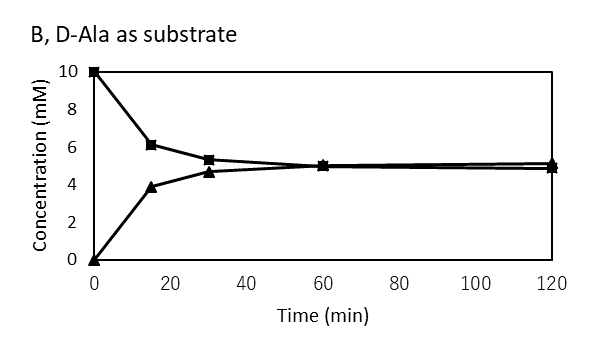


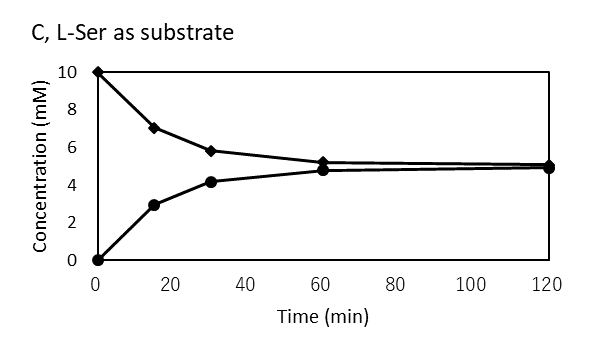

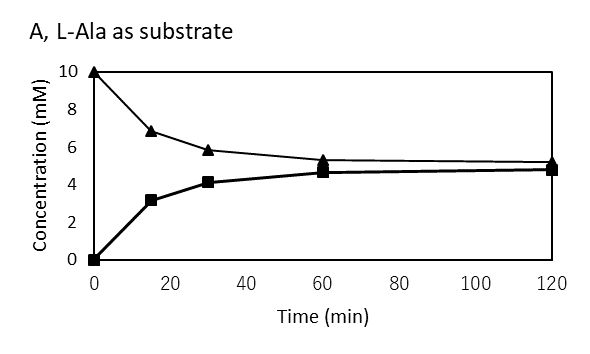

Supplement: Supplementary file 1 [file Data_Sheet_1.docx]
